# Pre-attack and pre-episode symptoms in cluster headache: a multicenter cross-sectional study of 327 Chinese patients

**DOI:** 10.1186/s10194-022-01459-z

**Published:** 2022-07-30

**Authors:** Ke Li, Shuping Sun, Zhanyou Xue, Sufen Chen, Chunyang Ju, Dongmei Hu, Xiaoyu Gao, Yanhong Wang, Dan Wang, Jianjun Chen, Li Li, Jing Liu, Mingjie Zhang, Zhihua Jia, Xun Han, Huanxian Liu, Mianwang He, Wei Zhao, Zihua Gong, Shuhua Zhang, Xiaoxue Lin, Yingyuan Liu, Shengshu Wang, Shengyuan Yu, Zhao Dong

**Affiliations:** 1 Department of Neurology, the First Medical Center, Chinese PLA General Hospital, 100853 Beijing, China; 2 Medical School of Chinese PLA, 100853 Beijing, China; 3 Department of Neurology, Suzhou Blue Cross Brain Hospital, Suzhou, China; 4 Department of Neurology, Changsha Central Hospital affiliated to University of South China, Changsha, China; 5 Department of Neurology, Xuchang Central Hospital Affiliated to Henan University of Science and Technology, Xuchang, China; 6 Department of Neurology, The Second Affiliated Hospital of Shandong First Medical University, Taian, China; 7 Department of Neurology, the Affiliated Yantai Yuhuangding Hospital of Qingdao University, Yantai, China; 8 Department of Neurology, Changchun Hospital of traditional Chinese medicine, Changchun, China; 9 Department of Neurology, General Hospital of Northern Theatre command, Shenyang, China; 10 Department of Neurology, Lishui Municipal Central Hospital, Lishui, China; 11 Department of Neurology, Jincheng General Hospital, Jincheng, China; 12 International headache center, Chinese PLA General Hospital, 100853 Beijing, China; 13 Institude of geriatrics, Beijing Key Laboratory of Aging and Geriatrics, National Clinical Research Center for Geriatrics Diseases, the 2nd Medical Center, Chinese PLA General Hospital, Beijing, China

**Keywords:** Cluster headache, Pre-attack symptoms, Pre-episode symptoms

## Abstract

**Background:**

There have been a few studies regarding the pre-attack symptoms (PAS) and pre-episode symptoms (PES) of cluster headache (CH), but none have been conducted in the Chinese population. The purpose of this study was to identify the prevalence and features of PAS and PES in Chinese patients, as well as to investigate their relationships with pertinent factors.

**Methods:**

The study included patients who visited a tertiary headache center and nine other headache clinics between January 2019 and September 2021. A questionnaire was used to collect general data and information about PAS and PES.

**Results:**

Among the 327 patients who met the CH criteria (International Classification of Headache Disorders, 3rd edition), 269 (82.3%) patients experienced at least one PAS. The most common PAS were head and facial discomfort (74.4%). Multivariable logistic regression analysis depicted that the number of triggers (OR = 1.798, *p* = 0.001), and smoking history (OR = 2.067, *p* = 0.026) were correlated with increased odds of PAS. In total, 68 (20.8%) patients had PES. The most common symptoms were head and facial discomfort (23, 33.8%). Multivariable logistic regression analysis showed that the number of triggers were associated with increased odds of PES (OR = 1.372, *p* = 0.005).

**Conclusions:**

PAS are quite common in CH patients, demonstrating that CH attacks are not comprised of a pain phase alone; investigations of PAS and PES could help researchers better understand the pathophysiology of CH.

## Introduction

Cluster headache (CH), the most common form of trigeminal autonomic cephalalgia, is the most painful primary headache, with an overall male-to-female preponderance and a lifetime prevalence of 124 per 100 000 [1]. It is characterized by strictly unilateral severe pain, accompanied by ipsilateral autonomic symptoms and/or restlessness [2, 3]. The low prevalence, lack of public awareness, and scarcity of medical experts specializing in CH all contribute to diagnostic delays, especially in China [4]. According to recent studies, some CH patients report pre-episode symptoms (PES) that begin days to weeks before the commencement of cluster episodes, as well as pre-attack symptoms (PAS) that begin minutes before the pain in individual attacks [5, 6]. Because PAS/PES occur before CH, indicating an early and a warning effect for headache attacks and cluster episodes, their identification and recognition may allow for earlier abortive and preventive treatment.

However, the prevalence of PAS exhibit considerable heterogeneity among countries and regions, ranges from 61.3 to 97.6% [5–11]. The Chinese population has not yet been examined for PAS and PES of CH. As a result, the purpose of this study was to look into the prevalence and features of PAS and PES in Chinese patients with CH.

## Methods

### Patients

CH patients were recruited from the International Headache Center, Department of Neurology of the Chinese PLA General Hospital, and nine other headache clinics between January 2019 and September 2021. All patients who had been diagnosed with CH, according to the ICHD-3 [2], were invited to participate in the study. Patients who agreed were then monitored by two qualified headache experts to exclude secondary and probable CH. The inclusion criteria were as follows: consistent with the ICHD-3 diagnostic criteria; headache in the cluster episodes; no abnormalities on physical examination (including fundus examination) and imaging examination (plain and contrast-enhanced brain magnetic resonance imaging); and ability to complete the questionnaire survey. The exclusion criteria were diagnosis of secondary headache; not able to distinguish their CH attacks from other types of headache.

### Data collection

Before enrollment, special training was provided to doctors at the clinics about consultation technique involved in the study (e.g. what are the symptoms that frequently rather than occasionally precede the headache, to ensure consistency of premonitory symptoms in the individual cluster patient) to ensure that they could accomplish the semi-structured questionnaire in a consistent manner. General data in the questionnaire included: demographic information (e.g., sex, age, height, weight, education level, occupation, long-term residence, smoking habits, and drinking habits); disease-related information (e.g., diagnosis, course of disease, incidence age, years of misdiagnosis, the family history of CH, and coexisting other types of headache); headache characteristics, including severity (visual analog scale rating from 0 to 10) and nature of headache, locations (frontal, parietal, occipital, temporal, orbital, retro-orbital, facial, nose, ear, teeth, and neck), frequency, attack duration (min), peak time of headache, circadian rhythm, and accompanying cranial autonomic symptoms (CAS); additional features (e.g., nausea, vomiting, photophobia, phonophobia, behaviors during attacks, and aggravation after activity); triggers and alleviating factors; and seasonality, frequency, and duration of the cluster episode. Information regarding PAS and PES was collected as following. The PAS were divided into three subtypes. General symptoms (29 items): yawning, irritability, anxiety and upset, overactivity, frustration, fatigue, dysesthesia, drowsiness, concentration changes, unwillingness to talk, loquacity, dysphasia, sensation of cold, dizziness, food craving, thirst, poor appetite, diarrhea, constipation, sweating, diuresis, nausea, palpitation, photophobia, phonophobia, osmophobia, edema, fidgeting, and other general symptoms; local discomfort symptoms (3 items): head and facial discomfort, ear swelling, and neck stiffness; CAS (8 items): lacrimation, conjunctival injection, nasal congestion and/or rhinorrhea, ptosis and/or miosis, and eyelid edema, and forehead and facial sweating. The PES was examined via open questions without specific symptoms, which were described by the patient and recorded by the doctors.

The average interval from the PAS to onset of headache attack (in minutes) and the PES to initiation of current episode (in days) were further asked.

### Statistical analysis

Statistical analyses were performed using SPSS (version 23.0; SPSS, Chicago, IL, USA) and the R Programming Language (version 3.6.2). Measurement data are expressed as means ± standard deviations or medians (interquartile ranges). Count data is expressed as numbers (percentages). Categorical variables were contrasted using the chi-squared test or Fisher’s exact test; continuous variables were compared using Student’s *t*-test or the Mann–Whitney U test. All the independent variables with *p*-values of less than 0.05 were selected for the multiple logistic regression analysis. A two-tailed *p* < 0.05 was indicated to indicate statistical significance. Linear fit was used to assess the correlation between variables.

## Results

### Demographics

**Fig. 1:**
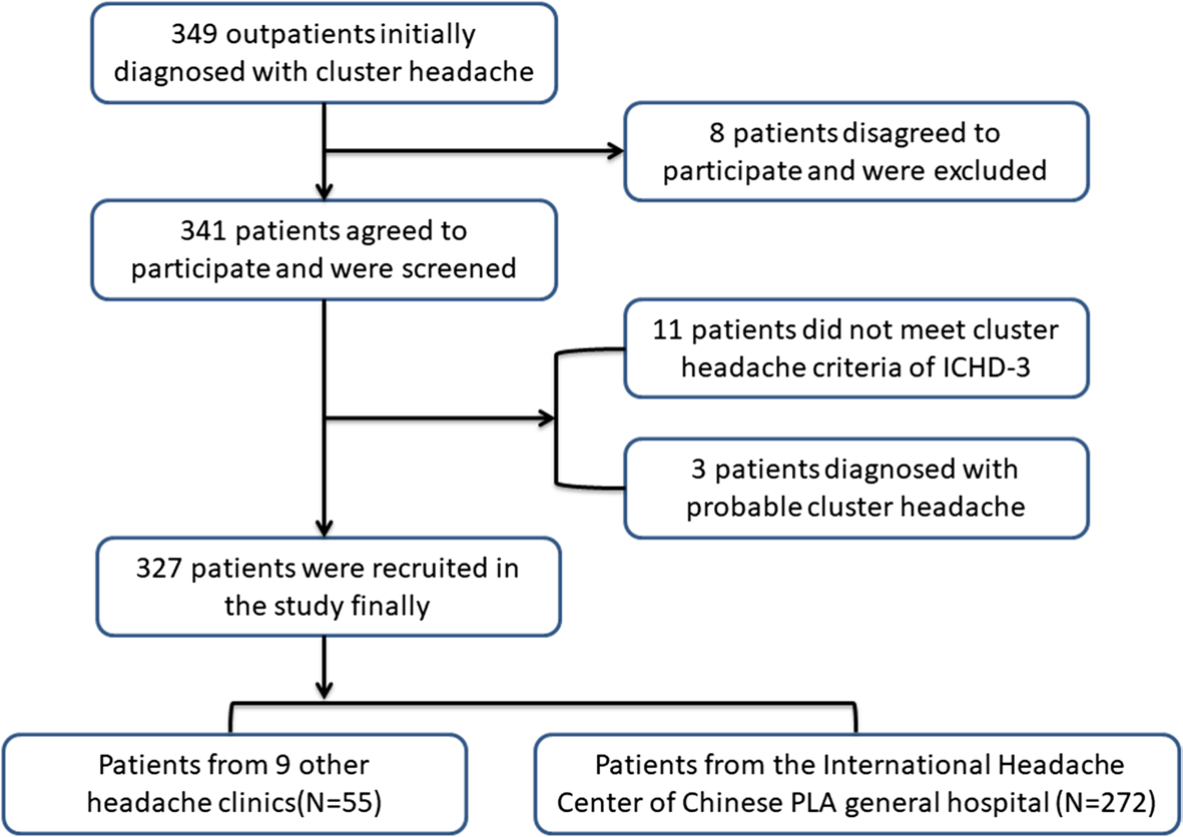
Flow chart of patient enrollment

Totally, 349 patients initially diagnosed with CH were invited to enroll in the study, and 341 patients agreed to participate. After a review of the patients’ information by two headache experts, 14 patients were excluded (Fig. 1). Finally, 327 patients were included in the study, of whom 318 were diagnosed with episodic CH and 9 were diagnosed with chronic CH. The study population consisted of 269 men and 58 women (a male-to-female [M:F] ratio of 4.6:1), with a median duration of 10 (interquartile range, 5.0–15.0) years (Table 1).

**Table 1:** Demographics and characteristics of patients with cluster headache, according to pre-attack and pre-episode symptoms

	Total (*N* = 327)	pre-attack symptoms		*p-* value	pre-episode symptoms		*p-* value
		None (*n* = 58)	Yes(*n* = 269)		None(*n* = 259)	Yes(*n* = 68)	
Age	33.0 (28.0–39.0)	31.0 (27.0–38.0)	33.0 (28.0–39.0)	0.210	32.0 (28.0–38.0)	34.0 (28.0–39.0)	0.141
Gender				0.734			0.091
Male	269 (82.3%)	47 (81.0%)	222 (82.5%)		218 (84.2%)	51 (75.0%)	
Female	58 (17.7%)	11 (19.0%)	47 (17.5%)		41 (15.8%)	17 (25.0%)	
Smoking history	144 (44.0%)	18 (31.0%)	126 (46.8%)	0.037*	110 (42.5%)	34 (50.0%)	0.324
Drinking history	109 (33.3%)	13 (22.4%)	96 (35.7%)	0.064	85 (32.8%)	24 (35.3%)	0.774
Diagnosis				0.722			0.915
eCH	318 (97.2%)	56 (96.6%)	262 (97.4%)		252 (97.3%)	66 (97.1%)	
cCH	9 (2.8%)	2 (3.4%)	7 (2.6%)		7 (2.7%)	2 (2.9%)	
Duration (years)	10.0 (5.0–15.0)	8.0 (2.8–15.0)	10.0 (5.0–15.0)	0.328	10.0 (5.0–15.0)	10.0 (7.0–15.0)	0.026*
Family history of CH	19 (5.8%)	2 (3.4%)	17 (6.3%)	0.414	15 (5.8%)	4 (5.9%)	0.996
Coexisting other types	47 (14.4%)	6 (10.3%)	41 (15.2%)	0.185	35 (13.5%)	12 (17.6%)	0.234
Number of headache locations	3.0 (2.0–4.0)	2.0 (1.0–3.0)	3.0 (2.0–4.0)	0.009*	3.0 (2.0–4.0)	3.0 (2.0–4.0)	0.362
VAS	9.0 (8.0–10.0)	8.5 (7.5–10.0)	9.0 (8.0–10.0)	0.025*	9.0 (8.0–10.0)	9.5 (8.5–10.0)	0.046*
Duration of episode				0.089			0.364
Less than 2 weeks	34 (10.4%)	12 (20.7%)	22 (8.2%)		30 (11.6%)	4 (5.9%)	
From 2 weeks to less than 1 month	77 (23.5%)	14 (24.1%)	63 (23.4%)		57 (22.0%)	20 (29.4%)	
From 1 to 2 months	164 (50.2%)	24 (41.4%)	140 (52.1%)		127 (49.0%)	37 (54.4%)	
More than 2 months	43 (13.1%)	5 (8.6%)	38 (14.1%)		37 (14.3%)	6 (8.8%)	
First experience of cluster	9 (2.8%)	3 (5.2%)	6 (2.2%)		8 (3.1%)	1 (1.5%)	
Frequency of episode				0.996			0.092
Less than 1 time/year	136 (41.6%)	24 (41.4%)	112 (41.6%)		108 (41.7%)	28 (41.2%)	
1 time/year	121 (37.0%)	22 (37.9%)	99 (36.8%)		101 (39.0%)	20 (29.4%)	
More than 1 time/year	70 (21.4%)	12 (20.7%)	58 (21.6%)		50 (19.3%)	20 (29.4%)	

*p＜0.05;Data are presented as medians (interquartile ranges) or numbers (percentages)

### Clinical characteristics

The duration of cluster attacks were 1–2 months in 50.2% of patients, 2 weeks to less than 1 month in 23.5%, more than 2 months in 13.1% and less than 2 weeks in 10.4%. For the cluster episode, 41.6% of patients had less than one per year, and the others had one (37.0%) or more than one (21.4%). In our cohort, 44.0% of patients had a positive history of tobacco exposure, and 33.3% of the surveyed patients stated they drank alcohol (Table 1).

### Pre-attack symptoms

Overall, 269 (82.3%) patients had PAS. Comparison of the demographic characteristics of patients with and without PAS revealed that more people with PAS smoked (*p* = 0.037). In comparing headache characteristics between patients with PAS and those without PAS, a higher number of headache locations (2.0 vs. 3.0; *p* = 0.009), higher visual analog scale rating (8.5 vs. 9.0; *p* = 0.025) were observed in patients with PAS. There were no significant differences between groups in terms of age, sex, drinking history, diagnosis, family history of CH, coexisting other types, disease duration, duration of episode or frequency of episode.

**Fig. 2:**
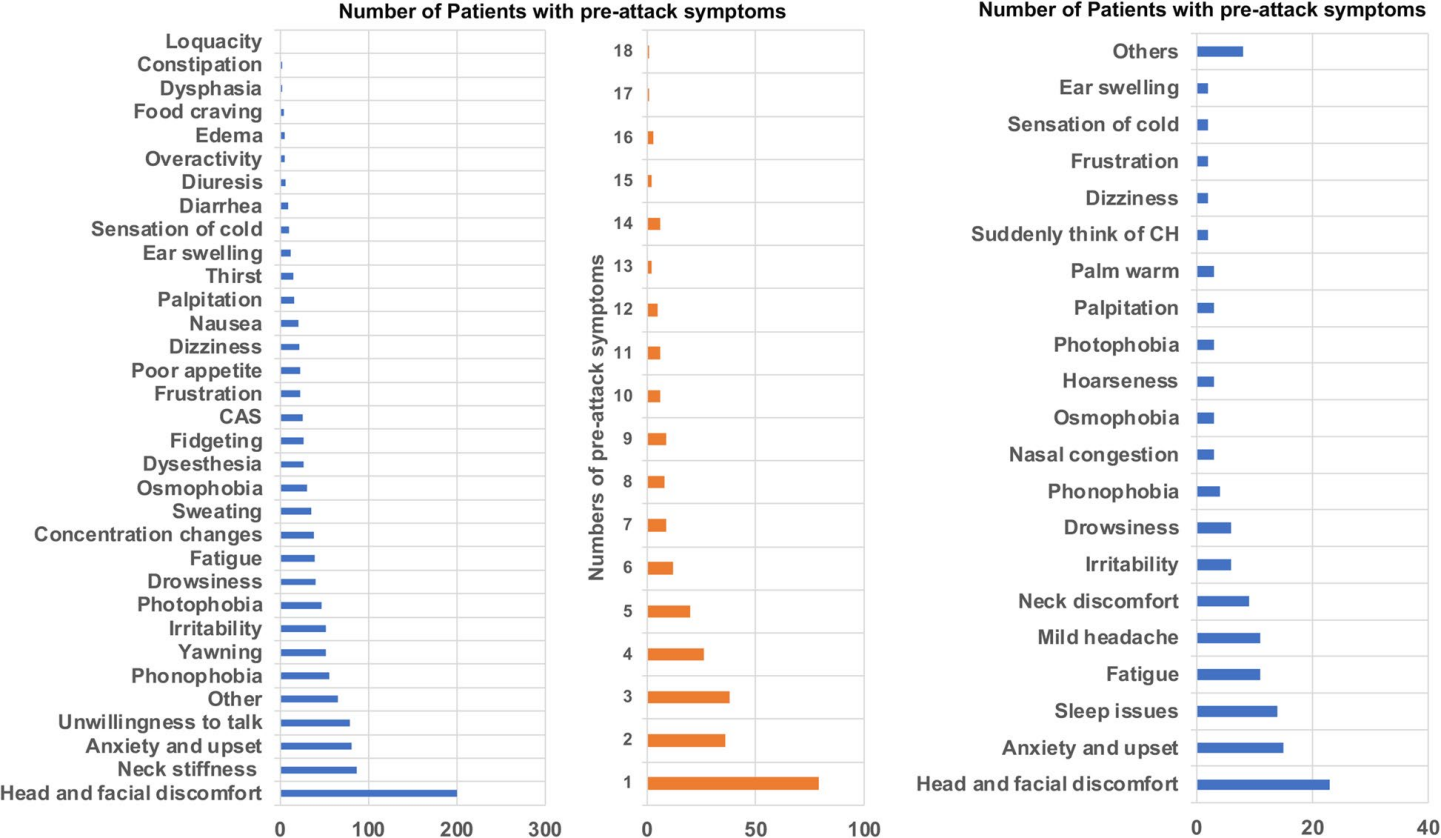
The left shows the number of patients with pre-attack symptoms; the middle shows patients experiencing different numbers of pre-attack symptoms simultaneously; the right shows the number of patients experiencing different pre-episode symptoms

**Fig. 3:**
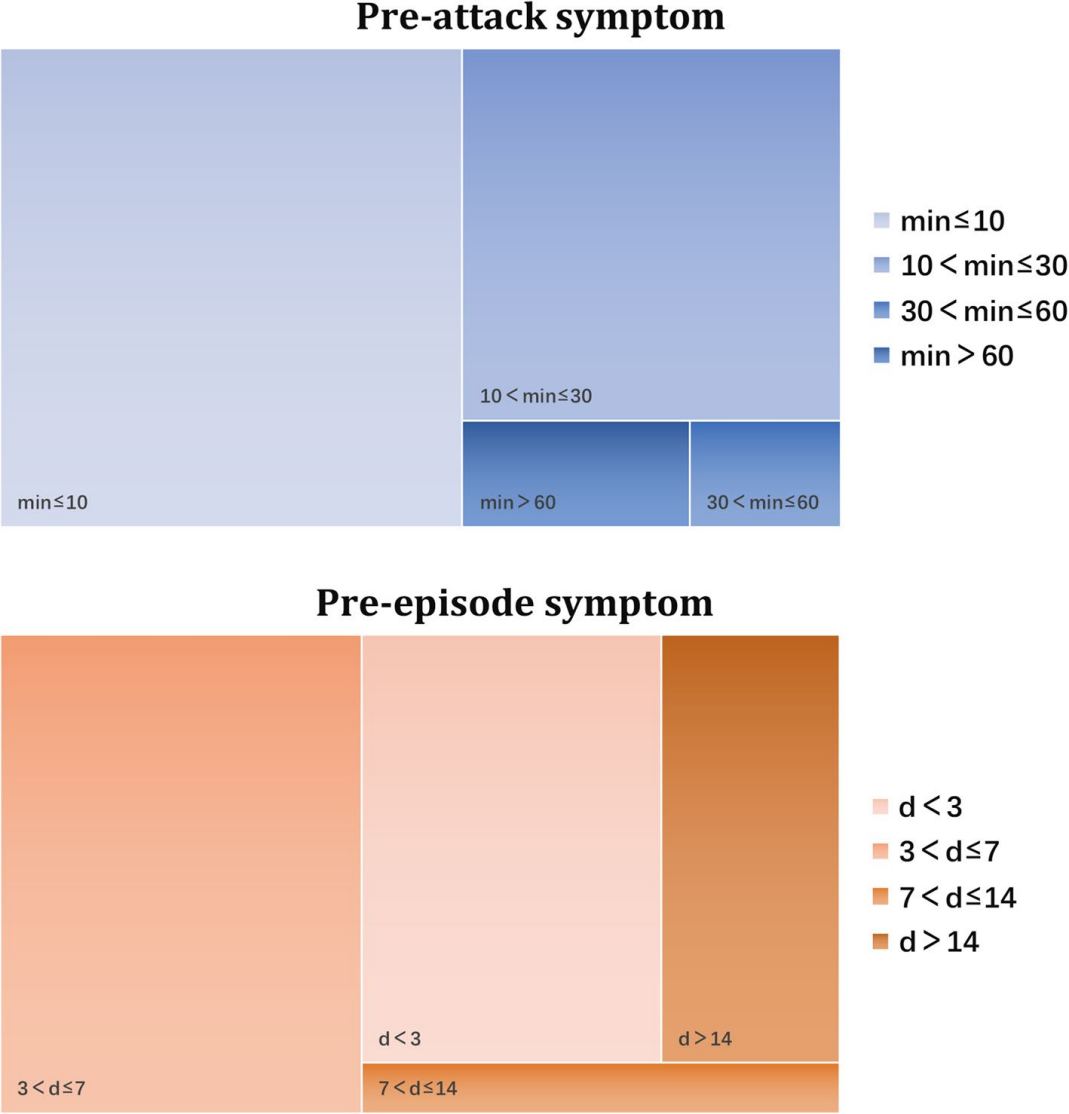
Durations of pre-attack and pre-episode symptoms before headache

Among patients with PAS, 29.4% of them reported experiencing one PAS, 13.4% of them reported experiencing two PAS, and 14.1% reported experiencing three PAS; the remaining 43.1% reported experiencing more than three symptoms (Fig. 2). The most common PAS was head and facial discomfort (74.4%), followed by neck stiffness (32.3%), anxiety and upset (30.1%), and was unwillingness to talk (29.4%). Other PAS were phonophobia (20.8%), yawning (19.3%), irritability (19.3%), photophobia (17.5%), drowsiness (14.9%), fatigue (14.5%), and changes in concentration (14.1%) (Fig. 2). The prevalence of subtypes was as follows: local discomfort symptoms, 82.5%; general symptoms, 65.8%; and CAS, 9.3%. With regard to the timing of PAS before headache, most patients had an interval of ≤ 10 min (149 cases, 55%), followed by 10–30 min (93 cases, 35%), > 60 min (15 cases, 6%), and 30–60 min (12 cases, 4%) (Fig. 3).

**Fig. 4:**
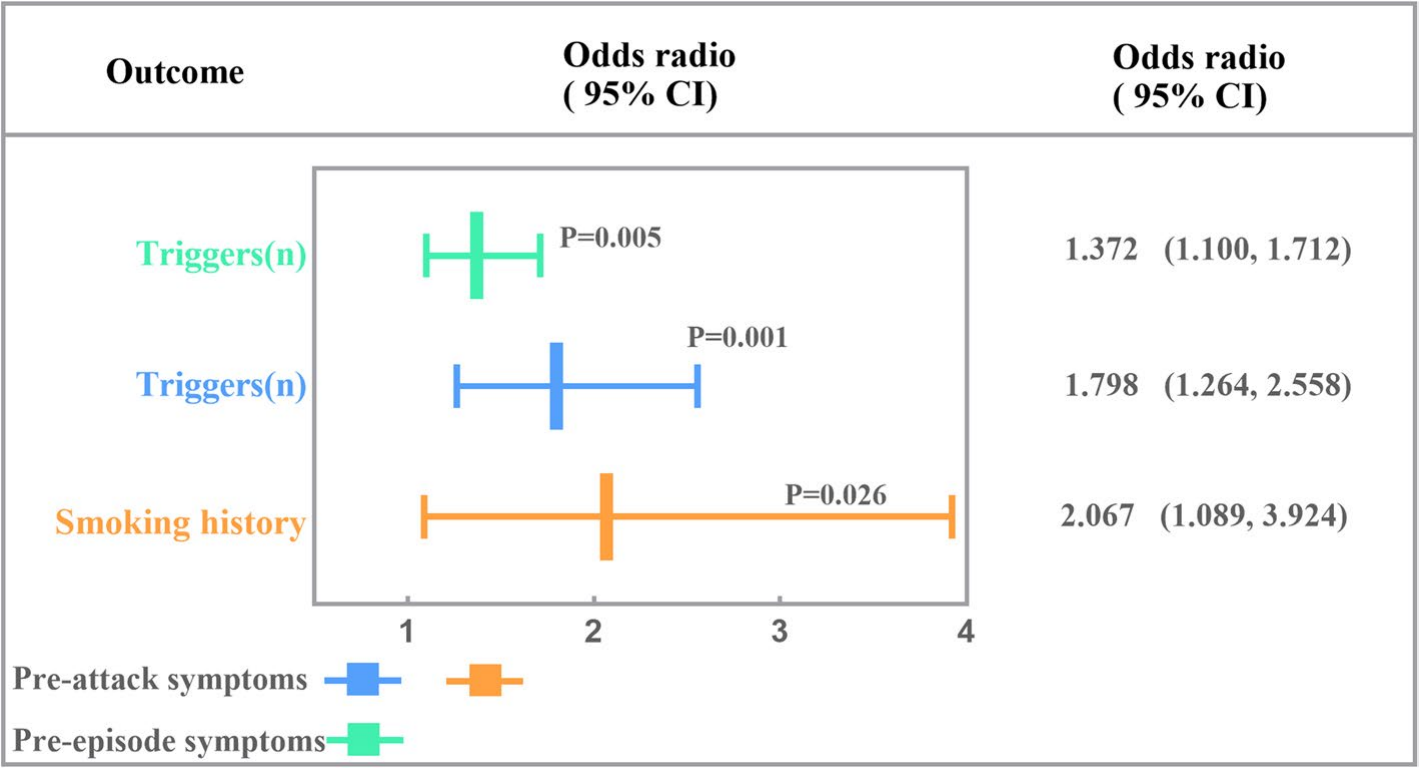
Multivariable logistic regression analyses to assess factors associated with PAS and PES

The factor associated with the presence of PAS in the univariable and multivariable logistic regression analyses was the number of triggers (OR = 1.798, 95% CI = 1.264–2.558, *p* = 0.001), and smoking history (OR = 2.067, 95% CI = 1.089–3.924, *p* = 0.026) (Table 2; Fig. 4).

**Table 2:** Univariable and multivariable logistic regression analyses to assess factors associated with PAS

	Univariable analysis			Multivariable analysis		
	OR	95% CI	*p*-value	OR	95% CI	*p-*value
Age	1.023	0.990–1.057	0.177			
Gender(male)	0.905	0.437–1.873	0.787			
Smoking history	1.958	1.069–3.588	0.030*	2.067	1.089–3.924	0.026*
Drinking history	1.921	0.987–3.737	0.055			
Diagnosis(eCH)	0.748	0.151–3.697	0.722			
Duration (years)	1.018	0.979–1.058	0.371			
Family history of CH	1.889	0.424–8.411	0.404			
Coexisting other types	1.558	0.629–3.864	0.338			
Number of headache locations	1.310	1.071–1.603	0.009*	1.204	0.980–1.478	0.077
VAS	1.277	1.025–1.592	0.030*	1.169	0.912–1.497	0.217
Duration of episode						
Less than 2 weeks	2.152	0.855–5.416	0.104			
From 2 weeks to less than 1 month	2.810	1.214–6.501	0.016*			
From 1 to 2 months	3.539	1.089–11.498	0.036*			
More than 2 months	0.957	0.201–4.557	0.955			
First experience of cluster	(Ref)		(Ref)			
Number of triggers	1.828	1.325–2.520	0.001*	1.798	1.264–2.558	0.001*
Number of mitigating factors	1.643	1.067–2.531	0.024*	1.340	0.842–2.132	0.217
Number of non-CAS symptoms	1.206	1.043–1.394	0.011*	1.151	0.975–1.360	0.097

*p < 0.05

To observe the distribution of smokers and non-smokers in the different types of PAS, we counted the number and percentage of smokers/non-smokers for each type of PAS in Table 3.

**Table 3:** The number and percentage of smokers/non-smokers for each type of PAS

Pre-attack symptom	Total (*N* = 269)	Smoking history		*P*-value
		No (*N* = 143)	Yes (*N* = 126)	
Head and facial discomfort	200 (74.3%)	104 (72.7%)	96 (76.2%)	0.070
Neck stiffness	87 (32.3%)	48 (33.6%)	39 (31.0%)	0.862
Anxiety and upset	81 (30.1%)	39 (27.3%)	42 (33.3%)	0.102
Unwillingness to talk	79 (29.4%)	36 (25.2%)	43 (34.1%)	0.033
Others	57 (21.2%)	35 (24.5%)	22 (17.5%)	0.363
Phonophobia	56 (20.8%)	26 (18.2%)	30 (23.8%)	0.114
Yawning	52 (19.3%)	30 (21.0%)	22 (17.5%)	0.784
Irritability	52 (19.3%)	23 (16.1%)	29 (23.0%)	0.063
Photophobia	47 (17.5%)	22 (15.4%)	25 (19.8%)	0.172
Drowsiness	40 (14.9%)	21 (14.7%)	19 (15.1%)	0.638
Fatigue	39 (14.5%)	20 (14.0%)	19 (15.1%)	0.530
Concentration changes	38 (14.1%)	19 (13.3%)	19 (15.1%)	0.431
Sweating	35 (13.0%)	13 (9.1%)	22 (17.5%)	0.018
Osmophobia	30 (11.2%)	13 (9.1%)	17 (13.5%)	0.144
Dysesthesia	26 (9.7%)	10 (7.0%)	16 (12.7%)	0.061
Fidgeting	26 (9.7%)	11 (7.7%)	15 (11.9%)	0.144
CASs	25 (9.3%)	14 (9.8%)	11 (8.7%)	0.997
Frustration	23 (8.6%)	7 (4.9%)	16 (12.7%)	0.011
Poor appetite	23 (8.6%)	9 (6.3%)	14 (11.1%)	0.199
Dizziness	22 (8.2%)	7 (4.9%)	15 (11.9%)	0.018
Nausea	21 (7.8%)	11 (7.7%)	10 (7.9%)	0.732
Palpitation	16 (6.0%)	6 (4.2%)	10 (7.9%)	0.127
Thirst	15 (5.6%)	9 (6.3%)	6 (4.8%)	0.747
Ear swelling	12 (4.5%)	5 (3.5%)	7 (5.6%)	0.309
Sensation of cold	10 (3.7%)	4 (2.8%)	6 (4.8%)	0.302
Diarrhea	9 (3.3%)	6 (4.2%)	3 (2.4%)	0.512
Diuresis	6 (2.2%)	1 (0.7%)	5 (4.0%)	0.050
Overactivity	5 (1.9%)	5 (3.5%)	0	0.046
Edema	5 (1.9%)	3 (2.1%)	2 (1.6%)	0.855
Food craving	4 (1.5%)	2 (1.4%)	2 (1.6%)	0.809
Dysphasia	2 (0.7%)	0	2 (1.6%)	0.110
Constipation	2 (0.7%)	1 (0.7%)	1 (0.8%)	0.865

**Fig. 5:**
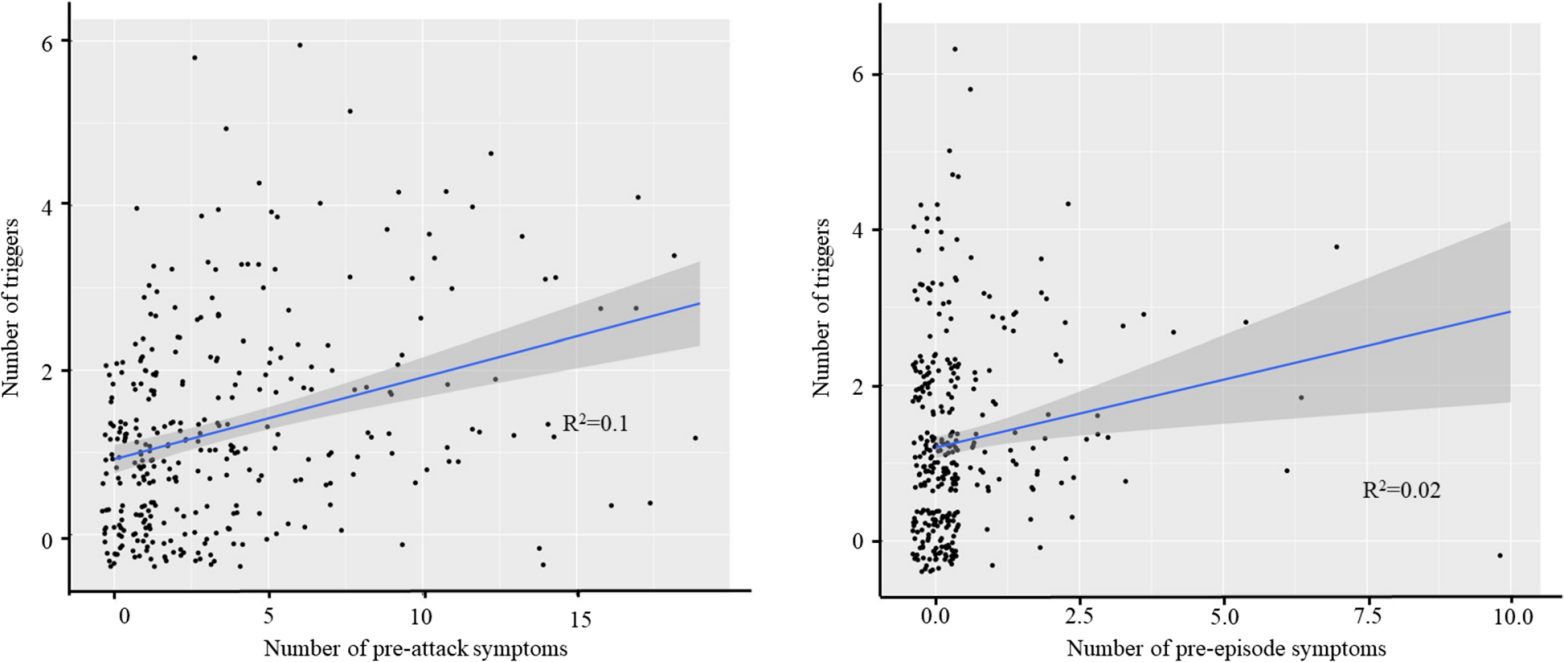
The left shows the correlation of number of triggers to number of PAS; the right shows the correlation of number of triggers to number of PES

We performed a linear fit, and the figure shows that the number of triggers and the number of PAS are highly correlated (Fig. 5).

### Pre-episode symptoms

In total, 68 patients (68/327, 20.8%) had PES. Among them, symptoms occurred ≤ 3 days before the cluster episode in 22 patients (32%), 3–7 days before in 29 patients (43%), 7–14 days before in 4 patients (6%), and > 14 days before in 13 patients (19%) (Fig. 3).

The most common symptoms were head and facial discomfort (23 cases, 33.8%), followed by anxiety and upset (15 cases, 22.1%), sleep issues (14 cases, 20.6%), and fatigue/mild headache (non-CH) (11 cases, 16.2%), neck discomfort (9 cases, 13.2%), irritability/drowsiness (6 cases, 8.8%), phonophobia (4 cases, 5.9%), nasal congestion/osmophobia/hoarseness/photophobia/palpitation/warm palms (3 cases, 4.4%), premonition of attacks/dizziness/frustration/sensation of cold/ear swelling (2 cases, 2.9%), shadow attacks (defined as a cluster-like episodes of milder pain and shorter duration/toothache/hyperesthesia/diuresis/redness of the eyes/food cravings/overactivity/constipation; 1 case, 1.5%) (Fig. 2).

One factor was associated with the presence of PES in univariable and multivariable logistic regression analyses: the number of triggers (OR = 1.372, 95% CI = 1.100–1.712, *p* = 0.005). There were no significant differences in demographic or disease-related information or headache characteristics between the groups (Table 4; Fig. 4).

**Table 4:** Univariable and multivariable logistic regression analyses to assess factors associated with PES

	Univariable analysis			Multivariable analysis		
	OR	95% CI	*p*-value	OR	95% CI	*p-*value
Age	1.025	0.997–1.054	0.081			
Gender(male)	1.772	0.932–3.369	0.081			
Smoking history	1.355	0.793–2.314	0.267			
Drinking history	1.117	0.637–1.957	0.700			
Diagnosis(eCH)	1.091	0.221–5.375	0.915			
Duration (years)	1.035	1.003–1.068	0.033*	1.020	0.986–1.056	0.255
Family history of CH	1.017	0.326–3.169	0.977			
Coexisting other types	1.371	0.669–2.812	0.389			
Number of headache locations	0.932	0.792–1.097	0.399			
VAS	1.273	1.001–1.619	0.049*	1.189	0.928–1.524	0.171
Duration of episode						
Less than 2 weeks	2.035	0.693–5.976	0.196			
From 2 weeks to less than 1 month	1.735	0.628–4.793	0.288			
From 1 to 2 months	0.784	0.207–2.968	0.720			
More than 2 months	0.725	0.074–7.125	0.783			
First experience of cluster	(Ref)		(Ref)			
Number of triggers	1.482	1.202–1.826	0.001*	1.372	1.100–1.712	0.005*
Number of mitigating factors	1.433	1.027–2.001	0.035*	1.245	0.876–1.771	0.222
Number of non-CAS symptoms	1.060	0.932–1.206	0.373			

*p < 0.05

The linear fit was performed to verify whether there was a correlation between the number of triggers and the number of PES. The results showed that the number of triggers was well correlated with the number of PES (Fig. 5).

## Discussion

As far as we know, this cross-sectional study on PES of CH had the largest sample size to date; it was also the first study to include PAS and PES. Our results showed that 82.3% of CH patients have PAS. The most common PAS was head and facial discomfort (74.4%), followed by neck stiffness (32.3%), anxiety and upset (30.1%), and unwillingness to talk (29.4%). The prevalence of PAS was significantly associated with the number of triggers and smoking history. Approximately 20% of CH patients had PES, the most common of which were head and facial discomfort (33.8%), anxiety and upset (22.1%), and sleep issues (20.6%). The appearance of symptoms before the cluster episode was independently associated with the number of triggers.

We agree with Blau et al., who suggested that local and painful symptoms may represent the accumulation of pain, rather than constituting specific PAS [7]. Therefore, we ignored these symptoms and included three categories in the questionnaire: local and painless sensory symptoms, CAS and general symptoms. The prevalence of PAS in our study (82.3%) was consistent with the prevalence described by Snoer et al. in Danish CH patients (83.3%) [6] and Cho et al. in Korean patients (71.3%) [10]. The most common PAS subtype consisted of local and painless sensory symptoms. Among these symptoms, head and facial discomfort (74.4%) and neck stiffness (32.3%) were reported by most patients. We defined head and facial discomfort as a dull, pulsation or throbbing sensation in the attack area, which is distinct from ache or pain; this was regarded as an attack build-up phenomenon [5, 7]. Among the 65.8% of patients who reported general symptoms before the attack, mood changes (e.g., anxiety and upset, unwillingness to talk, and irritability) were most common, suggesting activation of the hypothalamus and its regional distribution before the headache. Nociceptive stimulation of the trigeminal nerve is presumed to activate the parasympathetic nervous system and lead to the emergence of CAS [12]. However, autonomic phenomena but no pain before CH attacks were reported in 9.3% of cases; these findings suggested that the autonomic symptoms may not necessarily be driven by the trigeminal nerve [13].

Our study showed that the number of triggers and a history of smoking were all associated with an increased risk of PAS. The odds of developing PAS increased by 80% for each additional number of triggers, and by 100% for patients with a history of smoking. According to our results, an excessive number of triggers is related to a higher incidence of PAS and the number of triggers was positively correlated with the number of PAS. Our previous study explored the relationships between triggers and premonitory symptoms in migraine patients. There is speculation that some triggers may represent other premonitory symptoms, which can induce and accelerate the headache transformation process. Other triggers may induce brain dysfunction, making patients more prone to headache attacks [14]. Among CH patients in our study, the strong association between triggers and PAS also supports this speculation that triggers may prompt brain dysfunction in the pre-attack phase. Long-term cigarette smoking causes cadmium metabolite accumulation in the brain, altering the hypothalamic neurotransmitter pathways and hormone axis, according to previous animal research [15]. Rozen et al. showed that tobacco exposure proved to be a risk factor for chronic CH in a large clinical phenotypic research of CH patients in the United States, because the proportion of patients shifting from episodic to chronic CH is higher among individuals with tobacco exposure [16]. Persistent cigarette smoke leads to chronic low-dose cadmium exposure, which probably contributes to pathogenesis of CH [17]. In addition, our data demonstrated that smoking history is associated with increased odds of PAS; the data may also indicate the development of chronic susceptibility and hypothalamus-mediated reduction of the headache attack threshold in smokers. Further studies are required to determine whether the strong association between smoking history and the increased odds of PAS indicates that metabolites of cigarette smoke cause central nervous system damage (hypothalamus or other) before an attack; such studies should also investigate the specific underlying mechanisms.

PES was reported by one-fifth (21%) of our patients, which is lower than the rate of 86% reported in a prior Danish study [11]. This discrepancy is likely due to the fact that the Danish study employed closed questionnaires for specific issues, while we used open questionnaires. A recent study in Korea investigating the characteristics of PES among eCH patients showed that the upcoming cluster episode was predictable in 35.3% of the patients [18]. The results are almost identical to ours, despite the differences in questionnaire settings and sample sizes. Moreover, the PES of most patients appeared 3–7 days before the cluster episode in our study, which was similar to the timing in the Danish study (mean, 6.8 days). This time frame may provide more possibilities for early intervention in CH patients. However, further pertinent research is needed.

In patients with PES, the higher number of general symptoms such as local discomfort, anxiety and upset were similar to the PAS. However, 20% of patients reported sleep issues. The poor sleep quality during cluster episode reported by CH patients is most likely associated with frequent nocturnal attacks. Sleep issues, on the other hand, might persist during remission phases, suggesting the involvement of other contributing factors [19]. In a study of CH patients’ sleep disorders in the ictal and non-ictal phases, Lund et al. found no variations in sleep parameters between the two phases. However, when compared to healthy controls, sleep efficiency was lower, indicating that sleep issues in cluster episodes are slow and continuous processes [20, 21]. The sleep–wake cycle is regulated by the hypothalamus [22]. The appearance of sleep issues before cluster episodes may indicate the initiation and activation of central homeostasis regulation disorders.

### Strengths and limitations

The large sample size and multicenter study design supported the representativeness of the study sample. In addition, the questionnaire summarized all previous study items. However, our study has some limitations. First, recall bias is inevitable with a cross-sectional study design; Considering the severity of CH and the high frequency of attacks, most patients presumably had better memories of symptoms. Second, a cross-sectional design was adopted since prospective studies to observe the symptoms of patients without interventional measures do not meet ethical requirements. Moreover, because few research is able to provide preliminary data, the PES was collected via an open questionnaire. In addition, we did not ask patients about the prevalence of these symptoms without concurrent cluster headache or the frequency of their occurrence outside of the cluster episodes for patients who reported the presence of PAS/PES. When asking patients about their relevant symptoms, it is not excluded that some patients hold rather paranoid beliefs and attitudes about CH, and that tend to observe and report anything and symptoms regarding each attack or each cluster appearance in order to be able to explain or discover the disease’s cause. These potential biases should be taken into account. Finally, we did not retest the symptoms in a prospective study. Further longitudinal follow-up may increase the reliability of the data.

## Conclusions

This study showed that most CH patients had PAS. A greater number of triggers, and a history of smoking were associated with the increased odds of PAS. Approximately one-fifth of the patients had PES, and the number of triggers was associated with increased odds of PES. Analyses of PAS and PES can help to better understand the characteristics of the initial stage of CH, thus providing insights regarding the pathophysiological mechanisms of CH. By summarizing the rich clinical phenotypes, we can better predict the occurrence of headaches and provide evidence for further prevention and treatment.

## Data Availability

The datasets used and/or analysed during the current study are available from the corresponding author on reasonable request.

## Abbreviations

PAS: Pre-attack symptoms
PES: Pre-episode symptoms
CH: Cluster headache
CAS: Cranial autonomic symptoms
